# Association between excess weight and beverage portion size consumed in Brazil

**DOI:** 10.11606/S1518-8787.2018052000082

**Published:** 2018-02-07

**Authors:** Ilana Nogueira Bezerra, Eudóxia Sousa de Alencar

**Affiliations:** I Universidade Estadual do Ceará . Centro de Ciências da Saúde . Programa de Pós-Graduação em Nutrição e Saúde . Fortaleza , CE , Brasil; II Universidade de Fortaleza . Centro de Ciências da Saúde . Fortaleza , CE , Brasil

**Keywords:** Adult, Food Consumption, Beverages, Risk Factors, Overweight, epidemiology, Diet Surveys, Adulto, Consumo de Alimentos, Bebidas, Fatores de Risco, Sobrepeso, epidemiologia, Inquéritos sobre Dietas

## Abstract

**OBJECTIVE:**

To describe the beverage portion size consumed and to evaluate their association with excess weight in Brazil.

**METHODS:**

We used data from the National Dietary Survey, which included individuals with two days of food record aged over 20 years (n = 24,527 individuals). The beverages were categorized into six groups: soft drink, 100% fruit juice, fruit drink, alcoholic beverage, milk, and coffee or tea. We estimated the average portion consumed for each group and we evaluated, using linear regression, the association between portion size per group and the variables of age, sex, income, and nutritional status. We tested the association between portion size and excess weight using Poisson regression, adjusted for age, sex, income, and total energy intake.

**RESULTS:**

The most frequently consumed beverages in Brazil were coffee and tea, followed by 100% fruit juices, soft drinks, and milk. Alcoholic beverages presented the highest average in the portion size consumed, followed by soft drinks, 100% fruit juice, fruit drink, and milk. Portion size showed positive association with excess weight only in the soft drink (PR = 1.19, 95%CI 1.10–1.27) and alcoholic beverage groups (PR = 1.20, 95%CI, 1.11–1.29), regardless of age, sex, income, and total energy intake.

**CONCLUSIONS:**

Alcoholic beverages and soft drinks presented the highest averages in portion size and positive association with excess weight. Public health interventions should address the issue of portion sizes offered to consumers by discouraging the consumption of large portions, especially sweetened and low nutritional beverages.

## RESUMO

**OBJETIVO:**

Descrever o tamanho das porções de bebidas consumidas e avaliar sua associação com excesso de peso no Brasil.

**MÉTODOS:**

Utilizou-se dados do Inquérito Nacional de Alimentação, incluindo indivíduos com dois dias de registro alimentar, acima de 20 anos de idade (n = 24.527 indivíduos). As bebidas foram categorizadas em seis grupos: refrigerante; suco; refresco; bebida alcóolica; leite; e café ou chá. Estimou-se a porção média por ocasião de consumo para cada grupo e avaliou-se, por meio de regressão linear, a associação entre tamanho da porção por grupo e as variáveis idade, sexo, renda e estado nutricional. A associação entre tamanho da porção e excesso de peso foi testada por meio de regressão de Poisson, ajustada por idade, sexo, renda e ingestão total de energia.

**RESULTADOS:**

As bebidas com maior frequência de consumo no Brasil foram café e chá, seguidas dos sucos, refrigerantes e leite. As bebidas alcóolicas apresentaram maior média no tamanho da porção consumida, seguidas dos refrigerantes, sucos, refrescos e leite. O tamanho da porção mostrou associação positiva com excesso de peso somente no grupo dos refrigerantes (RP = 1,19; IC95% 1,10–1,27), e bebidas alcoólicas (RP = 1,20; IC95% 1,11–1,29), independentemente da idade, sexo, renda e ingestão total de energia.

**CONCLUSÕES:**

Bebidas alcoólicas e refrigerantes apresentaram as maiores médias no tamanho da porção e associação positiva com excesso de peso. Intervenções de saúde pública devem abranger a questão do tamanho das porções oferecidas aos consumidores, desencorajando o consumo de grandes porções, principalmente das bebidas adicionadas de açúcar e de baixo teor nutricional.

## INTRODUCTION

Food portion size can be defined as the actual amount of food that is placed on the plate, reflecting the choice of the consumer, restaurant, or food producer, or the amount of food or drink normally eaten or drank at the time of consumption ^2^ .

Some studies, both in developed countries ^12^ ^,^ ^14^ ^,^ ^20^ and in developing countries ^1^ ^,^ ^10^ ^,^ ^21^ , have shown an increase in the food portion size consumed, especially in relation to sweetened beverages. This increase in the amount of food consumed coincides with the increase in the prevalence of overweight and obesity in the population of several countries ^23^ .

Although excessive weight gain is caused by a complex network of multiple factors, the energy imbalance resulting from excessive energy intake and reduced energy expenditure is the proximal factor that best explains the development of overweight or obesity. The increase in portion sizes has been considered an important factor for this excessive weight gain because of its contribution to greater energy intake ^23^ ^,^ ^24^ . The mechanisms are still not well understood and the recommendations do not reflect the knowledge and complexity of the subject, but the literature suggests that adults substitute or ignore the signs of hunger and satiety when consuming large portions without energy compensation in subsequent meals ^2^ ^,^ ^4^ .

Despite the evidence that beverage portion size may be related to excessive weight gain, most studies on this subject have been carried out in developed countries, especially in the United States. In Brazil, only one study has evaluated the food portion sizes consumed, in a sample of adults of the city of São Paulo. The authors have found that large food portions from some groups with different energy densities, such as pizza, red meat, rice, snack foods, and soft drinks, are positively associated with excess weight ^13^ . However, there are no national studies that have estimated the beverage portion size consumed in a representative sample of the country and its relationship with excess weight.

The limitation of national data on the subject suggests a new focus of research and intervention aimed at subsidizing public health actions for changes in the environment in favor of preventing excess weight and the development of chronic non-communicable diseases related to poor diet. An analysis of the beverage portion size consumed in the country can also aid the evaluation of beverage consumption trends over the years in Brazil. The objective of this study is to describe the beverage portion size consumed and to evaluate its association with excess weight.

## METHODS

This is a cross-sectional study based on secondary data from the National Dietary Survey (INA), carried out together with the Household Budget Survey – POF (2008/2009).

The POF (2008/2009) was carried out from May 19, 2008, to May 18, 2009, with national coverage, including urban and rural areas and all income strata. It was conceptualized according to the concept of master sample, which the Brazilian Institute of Geography and Statistics (IBGE) has been adopting for all surveys with domiciliary sample from an Integrated System of Household Surveys, which corresponds to a set of census tracts based on the 2000 Demographic Census.

The tracts included in the master sample were geographically and statistically stratified, which allowed the generation of representative estimates for the Brazilian regions, urban and rural areas, capital, metropolitan region, and different income classes. The sectors tracts that make up the master sample were selected by sampling with probability proportional to the number of households per tract within each stratum.

The sampling plan used in the 2008/2009 POF was the conglomerate in two steps. The first step was the selection of master sample tracts by simple random sampling, amounting to 4,696 sectors. In the second step, the units sampled were the households, selected by simple random sampling within each of the tracts selected, amounting to 55,970 households investigated.

The INA was carried out in a sample of 24% of the households participating in the 2008/2009 POF, in which at least one resident over 10 years of age was interviewed (n = 34,003). For this study, we considered only the individuals who had two days of food record (n = 32,900). Individuals under 20 years of age (n = 7,342), pregnant women, and nursing mothers (n = 1,031) were excluded, amounting to a final sample of 24,527 individuals.

Food consumption data were collected using two food records on non-consecutive days, in which the individuals recorded all food and beverages consumed, the type of preparation, the amount consumed, and the time and place of the meals. Participants received guidance to complete the individual food consumption block from an information booklet containing illustrations of home measures to facilitate the correct filling of the amount of food consumed.

The records were reviewed by interviewers with survey questions if they found fewer than five items consumed over a day, avoiding the underreporting of participants. When no record was observed at intervals longer than three hours, the interviewer confirmed the lack of food intake at that time with the participant.

The amounts of food and beverages consumed in the reports in home measures were transformed into grams or milliliters for the calculation of the amount consumed of each item per informant, based on a table of home measures developed for INA. Details on the quantification of the food and beverages consumed can be found in an official IBGE publication ^a^ . For the estimation of the total energy intake of the individuals, we used the table of nutritional composition of the food consumed in Brazil ^b^ .

During the visitation period, the residents in the household were anthropometrically evaluated (weight and height). A portable electronic scale with 150 kg capacity and 100 g graduation was used to measure weight. In addition, a portable stadiometer with a retractable tape measuring up to 200 cm and a precision of 0.1 cm was used to measure height. The individuals were measured in a flat and well-lit place, without their shoes.

To evaluate the nutritional status of the population, we calculated the body mass index (BMI) from the weight divided by the height squared. The classification of the BMI of the adults followed the parameters of the World Health Organization: low weight (≤ 18.5 kg/m ^2^ ), normal weight (18.5 kg/m ^2^ to 24.9 kg/m ^2^ ), overweight (24.5 kg/m ^2^ to 29.9 kg/m ^2^ ), and obesity (≥ 30 kg/m ^2^ ). Because of the low percentage of individuals with low weight (2%), they were included in the normal weight category.

The data related to the characteristics of the household and residents that we considered in this study were age, sex, and household income *per capita* .

In order to estimate the household income, we added all the monetary and non-monetary income of the family members, and we obtained the household income *per capita* by dividing the household income by the number of residents living in it.

To analyze the beverage portions consumed, we considered the two days of food record. First, all beverages mentioned on the survey were grouped into six groups according to nutritional characteristics, type, and portion size: soft drinks, 100% fruit juices, fruit drink, alcoholic beverages, milk, and coffee and tea. Meal replacement drinks were excluded from this analysis.

The amount of beverages prepared from dilution was estimated based on the recommended dilution in the marketed product packaging. For example, the dilution of whole milk powder was calculated as follows: for every 200 ml of milk, two tablespoons of powdered milk (15 g) are recommended, that is, 100 ml of milk ready for consumption is made from 7.5 g of powder. The same transformation was performed for the items of skimmed milk powder, flavored milk powder, and soluble coffee. The other beverages prepared from powder, such as coffee powder and instant coffee, were already diluted into beverage portions in milliliters by the POF ^a^ table and did not require further treatment of the datum.

Then, we added the amount of beverages drank per food group at the time of consumption, and we calculated the average portion size consumed per individual from the sum of all the portions consumed by the individual divided by the number of occasions that these items were consumed for each beverage group.

We estimated the percentage of consumers in each beverage group, the median, and the average of the portion consumed of each group. We estimated the median portion of each group based on the entire population, while the average beverage portion size consumed was estimated only among consumers (individuals who recorded consumption of at least one beverage in the group in one of the two days of food record).

We tested the associations between the portion size consumed and the variables of age, sex, income, and nutritional status using linear regression models. The variables age, sex, income, and nutritional status were included in the model as independent variables, and portion size was included as dependent variable. The models to evaluate the relationship between portion size, income, sex, and nutritional status were adjusted for age. All statistical inferences were performed with 5% significance level.

We used Poisson regression models with robust variance to estimate the prevalence ratio (PR) of having excess weight (overweight and obese) according to the portion size consumed in each beverage group. For these analyses, we divided the portion size of each beverage group into three categories, considering non-consumers and the portion size normally marketed and consumed for individual portion: 350 mL (1 can) for soft drinks and alcoholic beverages, 200 mL (1 cup of tea) for coffee or tea, and 240 mL (1 average glass) for 100% fruit juices, fruit drink, and milk. Non-consumers were considered as the reference category. Initially, the models were adjusted for sex and age. Then, we added family income *per capita* and, finally, total energy intake.

The descriptive analyses were performed in the SAS software, version 9.1.3 (2003, SAS Institute, Cary, NC), and the Poisson regression models were developed in Stata software, version 13.0 (StataCorp. College Station, TX:2013), considering the sample weights of the INA and the effect of the study design.

## RESULTS

The study population had average age of 43.2 years, eight years of study, and average family income *per capita* of R$1,013.5. Half of the population was female (50.9%) and presented excessive weight (overweight and obese).

The most frequently consumed beverages in Brazil were coffee and tea, followed by 100% fruit juices, soft drinks, and milk, while the groups that presented the lowest consumption frequency were fruit drink and alcoholic beverages ( Table 1 ).

**Table 1:** Description, frequency (%), and 95% confidence interval (95%CI) of beverage consumption. Brazil, 2008/2009.

Beverage group	Description	Frequency (%)	95%CI
Soft drink	Soft drink, diet, light, energy drink, sports drink	35.4	34.0–36.8
100% fruit juice	Natural, pure or sweetened fruit juice	45.3	43.9–46.7
Fruit drink	Industrial, sweetened or sugar-free powdered juice	11.1	10.2–12.0
Alcoholic beverage	Beer, wine, liqueur, mixed alcoholic beverage	8.3	7.6–9.0
Milk	Whole milk, skimmed milk, semi-skimmed milk, pasteurized milk, chocolate milk, flavored milk, fermented milk, milk-based fruit vitamin	31.3	30.1–32.5
Coffee or tea	Coffee with or without sugar, cappuccino, coffee with milk, tea with or without sugar, diet or light	89.9	89.1–90.8

Regarding the portion consumed, the beverage group that presented the highest median was alcoholic beverages (525 mL) followed by soft drinks (280 mL). There was no difference between the median value of the 100% fruit juice, fruit drink, and milk groups (240 mL). The lowest median was found for the coffee or tea group (172.5 mL).

The alcoholic beverage group presented the highest average in the portion consumed at the time of consumption (710.8 mL, 95%CI 658.9–762.7), followed by the soft drink (308.2 mL, 95%CI 301.8–314.6), 100% fruit juice (264.5 mL, 95%CI 261.1–267.8), fruit drink (259.8 mL, 95%CI 251.8–267.8), milk (238.7 mL, 95%CI 235.2–242.3), and coffee or tea groups (177.3 mL, 95%CI 173.5–181.0). These averages increased with income only in the soft drink and 100% fruit juice groups ( Table 2 ).

**Table 2:** Averages and 95% confidence interval (95%CI) of the beverage portion size consumed (in mL), according to income quartiles and educational level. Brazil, 2008/2009.

Beverage group	Family income *per capita*								p*

	1st quartile		2nd quartile		3rd quartile		4th quartile		
			
	Average	95%CI	Average	95%CI	Average	95%CI	Average	95%CI	
Soft drink	298.7	288.1–309.3	299.9	289.3–310.4	317.3	302.5–332.0	310.7	299.7–321.6	0.006
100% fruit juice	258.0	253.1–262.9	262.4	256.1–268.8	267.8	261.6–273.9	267.6	260.5–274.7	0.002
Fruit drink	260.0	245.3–274.6	256.6	244.3–268.9	258.5	238.9–278.0	265.9	253.9–277.9	0.270
Alcoholic beverage	788.1	638.3–937.9	742.6	643.7–841.5	706.0	608.3–803.8	675.9	590.8–761.0	0.530
Milk	243.7	237.6–249.9	237.3	230.5–244.2	239.4	232.4–246.4	236.0	229.2–241.1	0.390
Coffee or tea	178.7	172.8–184.5	175.8	169.6–181.9	180.3	174.2–186.4	174.1	165.4–182.8	0.780

* p-value of the trend.

We observed that men reported higher averages of total consumption than women in all beverage groups. Portion size consumed decreased linearly with increasing age in the groups of soft drinks, 100% fruit juice, fruit drink, and milk, in both sexes. Among the male population, the same trend was observed for alcoholic beverages ( Table 3 ). Comparing the age groups, we observed higher consumption of soft drinks, 100% fruit juice, fruit drink, and alcoholic beverages among men aged 20–40 years and older adults (≥ 60 years) and among men aged 40–60 years and older adults. Among women, we observed differences in the 20–40 year old and older adult categories for the soft drink, 100% fruit juice, and fruit drink groups. Women aged 20–40 years also had a higher average in the portion size of soft drink consumption compared to those aged 40–60 years, and those aged 40–60 years had higher averages than female older adults.

**Table 3:** Averages and 95% confidence interval (95%CI) of the beverage portion size consumed (in mL), according to sex and age group. Brazil, 2008/2009.

Beverage group	Men				Women			
	
	Total	20–40 years	40–60 years	> 60 years	Total	20–40 years	40–60 years	> 60 years
							
	Average (95%CI)	Average (95%CI)	Average (95%CI)	Average (95%CI)	Average (95%CI)	Average (95%CI)	Average (95%CI)	Average (95%CI)
Soft drink	334.9 (325.3–344.5)	345.2 (331.9–358.6)	328.3 (313.9–342.7)	290.6 (264.7–316.5) ^a^	279.9 (274.1–285.8) ^b^	293.2 (284.6–301.8)	271.9 (262.9–280.9)	245.2 (236.3–254.0) ^a^
100% fruit juice	281.1 (275.9–286.3)	290.5 (282.9–298.2)	276.7 (269.6–283.8)	254.5 (246.2–262.9) ^a^	249.2 (246.1–252.3) ^b^	254.2 (249.7–258.8)	245.8 (241.1–250.5)	240.9 (233.8–247.9) ^a^
Fruit drink	276.0 (264.7–287.3)	287.4 (271.2–303.6)	272.3 (255.9–288.8)	239.8 (224.2–255.4) ^a^	243.9 (235.5–252.2) ^b^	255.5 (241.7–269.3)	236.4 (226.9–245.7)	224.9 (209.3–240.5) ^a^
Alcoholic beverage	774.6 (711.8–837.4)	849.0 (751.9–946.2)	775.7 (679.8–871.6)	449.9 (370.1–529.8) ^a^	524.5 (453.2–595.7) ^b^	549.2 (466.4–631.9)	524.3 (417.1–631.5)	386.9 (254.3–519.5)
Milk	253.6 (247.7–259.4)	262.2 (253.8–270.6)	249.1 (238.9–259.2)	233.4 (224.0–242.8) ^a^	226.2 (222.4–229.7) ^b^	229.7 (224.9–234.4)	225.2 (218.8–231.4)	219.8 (212.0–227.6) ^a^
Coffee or tea	183.5 (178.8–188.2)	183.9 (177.4–190.2)	183.7 (176.5–190.9)	182.1 (173.4–190.8)	171.3 (167.5–175.2) ^b^	173.2 (167.6–178.8)	172.5 (167.4–177.6)	165.0 (157.7–172.3)

^a^ Statistically significant linear trend (p < 0.05).

^b^ Statistically significant differences between the sexes (p < 0.05).

The analyses stratified by nutritional status showed that, with increasing BMI levels, portion size increased in the soft drink and alcoholic beverage groups among men, and normal weight men had a higher average in the soft drink portion size compared to obese men. The presence of overweight and obesity among the female population did not influence significant changes in the portion size consumed, except for the coffee or tea group ( Table 4 ).

**Table 4:** Averages and 95% confidence interval (95%CI) of the beverage portion size consumed (in mL), according to sex and nutritional status. Brazil, 2008/2009.

Group of beverages	Man				Woman			
	
	Eutrophic	Overweight	Obese	p*	Eutrophic	Overweight	Obese	p*
					
	Average (95%CI)	Average (95%CI)	Average (95%CI)		Average (95%CI)	Average (95%CI)	Average (95%CI)	
Soft drink	318.6 (308.1–329.0)	346.4 (328.4–364.4)	353.8 (334.0–377.7)	< 0.0001	281.8 (273.4–290.1)	277.0 (267.2–286.8)	279.6 (266,8–292.4)	0.343
100% fruit juice	281.8 (275.4–288.2)	281.2 (271.5–290.9)	278.8 (269.0–288.6)	0.617	247.8 (243.5–252.0)	249.3 (244.7–253.9)	254.0 (245.5–262.4)	0.040
Fruit drink	279.5 (261.7–297.3)	265.8 (251.9–279.7)	295.7 (271.0–320.5)	0.675	237.3 (227.2–247.5)	250.0 (233.7–266.3)	253.1 (233.5–272.8)	0.030
Alcoholic beverage	711.9 (632.5–791.4)	765.7 (666.6–864.9)	973.5 (781.4–1165.7)	0.002	555.2 (430.2–680.2)	501.3 (424.1–578.5)	471.1 (363.0–579.1)	0.541
Milk	255.8 (247.6–264.0)	251.7 (242.3–261.0)	251.4 (236.7–266.1)	0.752	228.9 (224.2–233.6)	223.1 (216.8–229.4)	222.1 (212.8–231.4)	0.312
Coffee or tea	180.0 (174.3–185.7)	184.4 (178.1–190.7)	195.3 (178.9–211.6)	0.050	166.8 (162.0–171.6)	174.0 (168.2–179.8)	179.3 (171.0–187.7)	0.001

* p-value of the trend adjusted for continuous age.

Only the size of the soft drink and alcoholic beverage portion showed a positive association with excess weight (overweight and obesity) and this relationship was independent of age, sex, income, and total energy intake ( Table 5 ).

**Table 5:** Prevalence ratio (PR) and 95% confidence interval of excess weight associated with beverage portion size consumed. Brazil, 2008/2009.

Beverage group	PR adjusted for age and sex (95%CI)	PR adjusted for age, sex, and income (95%CI)	PR adjusted for age, sex, income, and total energy (95%CI)
Consumption of soft drink			
No	1.00	1.00	1.00
< 350 mL	1.06 (1.00–1.11)	1.05 (1.00–1.11)	1.05 (1.00–1.11)
≥ 350 mL	1.20 (1.11–1.28)	1.19 (1.11–1.28)	1.19 (1.10–1.27)
Consumption of 100% fruit juice			
No	1.00	1.00	1.00
< 240 mL	1.02 (0.94–1.11)	1.02 (0.94–1.11)	1.01 (0.93–1.10)
≥ 240 mL	1.01 (0.97–1.06)	1.01 (0.97–1.06)	1.01 (0.96–1.05)
Consumption of fruit drink			
No	1.00	1.00	1.00
< 240 mL	0.89 (0.77–1.02)	0.89 (0.77–1.03)	0.89 (0.77–1.03)
≥ 240 mL	0.99 (0.92–1.06)	0.99 (0.92–1.06)	0.99 (0.92–1.06)
Consumption of alcoholic beverage			
No	1.00	1.00	1.00
< 350 mL	0.93 (0.80–1.08)	0.92 (0.79–1.08)	0.92 (0.79–1.07)
≥ 350 mL	1.21 (1.12–1.31)	1.20 (1.12–1.30)	1.20 (1.11–1.29)
Consumption of milk			
No	1.00	1.00	1.00
< 240 mL	0.94 (0.88–1.01)	0.94 (0.97–1.01)	0.94 (0.87–1.01)
≥ 240 mL	0.97 (0.92–1.02)	0.96 (0.91–1.02)	0.96 (0.91–1.01)
Consumption of coffee or tea			
No	1.00	1.00	1.00
< 200 mL	0.97 (0.89–1.05)	0.98 (0.90–1.06)	0.98 (0.90–1.06)
≥ 200 mL	1.04 (0.96–1.13)	1.05 (0.96–1.14)	1.05 (0.96–1.14)

## DISCUSSION

The findings of this study corroborate the hypothesis that the soft drink and alcoholic beverage portion size is positively associated with excess weight. The identification of the beverage portion size consumed in representative samples of the population is a necessary step to define adequate and country-specific dietary recommendations. This study reinforces the evidence of public health efforts to contain the obesity epidemic, suggesting a new look at behavioral risk factors that may be involved in excessive weight gain and that are modifiable. This is the first study to describe the beverage portion size consumed in Brazil in a representative sample of the country.

In Brazil, the beverage group most frequently consumed by the population was coffee or tea, which presented a smaller portion size at the time of consumption. On the other hand, although alcoholic beverages had the lowest frequency of consumption, they represent the largest portion size at the time of consumption, regardless of age and sex. The average portion size exceeds 700 mL, therefore exceeding the maximum recommended consumption proposed by the Brazilian Society of Cardiology for men (625 mL of beer or 312.5 mL of wine or 93.7 mL of distilled beverage) and women (312.5 mL beer or 156.25 mL of wine or 46.85 mL of distilled beverage) ^19^ .

The consumption of large alcoholic beverage portions not only favors higher energy intake (7 kcal/mL), but it is also associated with increased blood pressure and cardiovascular mortality in general. In addition, the consumption of large doses for long periods of time or acutely is related to cognitive dysfunction and may cause brain damage ^19^ ^,^ ^25^ .

We also highlight that ethanol consumption is much higher in the younger population. The exposure of young persons to health risk behaviors has become the target of scientific research as such behaviors can be incorporated early into the lifestyle of individuals ^5^ . Data showing the occurrence of health risk behaviors in young adults and their associated factors can help identify groups at risk and monitor the health levels of these individuals, as well as subsidize early intervention strategies to avoid the incorporation of these habits ^25^ .

Fifty-six percent of adults reported 100% fruit juice and soft drink consumption in one of the two days of the survey. Although 100% fruit juice and fruit drink suggest a healthier option compared to soft drinks, they are often consumed with added sugar. Most of the individuals (85%) who reported 100% fruit juice and fruit drink consumption reported using sugar to sweeten the beverages consumed. Another important issue to be considered is the consumption of processed juices which, for the most part, have high amounts of sugar in their composition. However, the relation between 100% fruit juice and fruit drink consumption with excess weight was not significant in this study.

As for soft drinks, although the frequency of consumption was lower than that of 100% fruit juices and fruit drink, the average portion is 17% higher than the juice group when chosen for consumption. In Mexico, this difference is even greater, as the size of portion of soft drinks is five times greater than the portion of 100% fruit juice ^21^ . This data shows the importance of not only the choice of the beverage but also of the amount drank at the time of consumption.

Evidence in developed countries suggests that the average size of the soft drink portion consumed increased from 387.4 mL to 588.5 mL between 1977 and 1998 and to 828 mL in 2004 ^3^ ^,^ ^9^ . Observing the reported trends on domestic availability of soft drinks in the country ^7^ , we believe that the soft drink portion consumed has also increased over the years in Brazil.

The concern with the consumption of soft drinks and sweetened juices and fruit drink is that the consumption of sweetened beverages is positively associated with the gain of body weight in children and adults. In addition, it is directly related to the increase in triglycerides, visceral adipose tissue, subcutaneous fat, and liver fat. The mechanisms by which these beverages interfere with health remain under discussion. It is believed that the high sugar content in sweetened beverages favors low satiety without compensation in the energy intake in subsequent meals, contributing with excessive weight gain by the increased energy intake ^9^ .

Men presented a higher consumption portion in all beverage groups when compared to the portion size consumed by women, corroborating the findings in the American and Mexican population for the consumption of soft drinks and alcoholic beverages ^21^ ^,^ ^c^ .

Among men, the effect of age on the reduction in portion size can be observed in all groups except for coffee and tea. Among women, we observed reduced portion size for the soft drink, 100% fruit juice, fruit drink, and milk groups with increasing age; this behavior can be seen as positive given the amount of sugar present in soft drinks, 100% fruit juices, and fruit drink. On the other hand, this fact draws attention to the greater portion of soft drinks consumed by younger adults. As discussed earlier, exposure of young persons to health risk behaviors leads to unhealthy lifestyles and poor eating habits in the future ^5^ . Regarding milk, although some studies show that its consumption may improve bone integrity ^18^ , there is still insufficient evidence that milk consumption protects against fractures, and it may be also associated with higher mortality ^8^ ^,^ ^11^ .

Considering nutritional status, we observed a linear increase in the portion size consumed for the soft drink and coffee or tea groups with increasing BMI among women and for the soft drink and alcoholic beverage groups among men. The supply of larger food portions leads to increased energy intake and some experimental studies have already shown no calorie compensation in subsequent meals, even after the high energy intake from large food portions. In addition, this effect is maintained for several days ^17^ .

This lack of caloric compensation appears to be greater in beverages, especially in those with added sugars, such as soft drinks. In Brazil, an analysis of the INA has found that the consumption of sweetened beverages increased by 158 kcal, 191 kcal, and 180 kcal the energy coming from breakfast, lunch, and dinner, respectively ^6^ .

In addition, soft drinks and alcoholic beverages can be vehicles of excessive energy consumption as they can lead to an increased intake of other types of food of high energy density and low nutritional density. Alcoholic beverages are also frequently consumed on special occasions, such as parties and barbecues, which last for a long time and lead to the consumption of larger amounts of food. Soft drinks and alcoholic beverages were the only groups that showed a positive association with excess weight in this study. The effect of the soft drink portion on excess weight has also been evidenced in the study by Pereira et al. ^13^ in the adult population of the city of São Paulo (OR = 1.016, 95%CI 1.004–1.028), but the authors emphasize that the portion size of other high energy food, such as pizza, red meat, and snacks, also contributes with excessive weight gain.

This study has as a strength the analysis of a little evaluated characteristic of food consumption in national studies, that is, the beverage portion size consumed, which can significantly affect the excessive consumption of calories and contribute with excessive weight gain. In addition, the data refer to a population sample of the country, covering all regions and different socioeconomic levels of Brazil. We highlight the contribution of this study to the formulation of public policies aimed at dietary changes, offering an opportunity for interventions that contribute with a reduction in calorie intake.

Some countries have sought to control the consumption of beverages, focusing on sweetened beverages, using taxation, reduced availability in schools, restrictions on marketing for children, public awareness campaigns, and positive and negative labeling on the packaging; however, little has been done specifically to regulate the size of the portions offered ^16^ . In New York, a 2012 regulatory measure limited the sweetened beverage portion size sold in food service and facilities, but law enforcement has encountered barriers ^15^ .

In Brazil, there are no policies aimed at reducing portion size. This study contributes to reinforce the view on an important subject that can favor the reduction in the total caloric intake affecting the maintenance of a healthy weight. However, the results should be viewed with caution, since the estimation of the amount of food consumed is an important source of error in dietary surveys. In this work, the consumption data come from the INA, which had food records as the collection method. Food records are prospective methods, in which individuals record the food and the portion consumed, and it is considered as accurate in the estimation of portion size, avoiding underreporting and memory bias ^22^ .

Another limitation of this study is that beverages represent food items often overlooked in dietary surveys. To avoid underreporting, the INA has incorporated the multiple-step method in data collection by asking survey questions on beverage consumption, but the impact of this bias on portion size estimation is unknown. An important issue to be considered is the possible modification in the portion size consumed on weekends. The records of the INA could be randomly made on any day of the week, as long as the days were not consecutive, but most of the records (91.7%) were performed on weekdays. The difference in portion size on weekdays is beyond the scope of this article.

In conclusion, the soft drink and alcoholic beverage portion size showed a positive association with excess weight. Although options considered as healthier, such as 100% fruit juices, are more frequently consumed than soft drinks, the portion size of this latter option is greater when chosen for consumption. Since the increase in portion size may contribute with excessive calorie intake and consequently excess weight, public health interventions should discourage the consumption of large portions, especially for sweetened and low-nutritional beverages. Other studies that address the portion size of other types of food and that consider size differences according to the days of the week are necessary for a better understanding of the eating behavior of individuals regarding the portion sizes consumed and the relationship between portion size and body weight.

## Footnotes

a Instituto Brasileiro de Geografia e Estatística. Pesquisa de Orçamentos Familiares 2008-2009: análise do consumo alimentar pessoal no Brasil. Rio de Janeiro: IBGE; s.d. [cited 2016 Feb 7]. Available from: https://ww2.ibge.gov.br/home/estatistica/populacao/condicaodevida/pof/2008_2009_analise_consumo/default.shtm

b Instituto Brasileiro de Geografia e Estatística. Pesquisa de Orçamentos Familiares 2008-2009: tabelas de composição nutricional dos alimentos consumidos no Brasil. Rio de Janeiro: IBGE; 2011 [cited 2016 Feb 7].Available from: https://biblioteca.ibge.gov.br/visualizacao/livros/liv50002.pdf

c LaComb RP, Sebastian RS, Enns CW, Goldman JD. Beverage choices of U.S. adults: what we eat in America, NHANES 2007–2008. Beltsville: Food Surveys Research Group; 2011 [cited 2016 Feb 7]. (Dietary Data Brief, 6). Available from: https://www.ars.usda.gov/ARSUserFiles/80400530/pdf/DBrief/6_beverage_choices_adults_0708.pdf
